# Potential gastrointestinal Behcet’s disease flare after treatment with anti-interleukin 17a therapy

**DOI:** 10.1186/s41927-023-00344-9

**Published:** 2023-08-08

**Authors:** Frances Sze Kei Sun, Nicole Sau Yan Chiu, Ho Yin Chung

**Affiliations:** 1 Division of Rheumatology and Clinical Immunology, Department of Medicine, The University of Hong Kong, Hong Kong, China; 2 Division of Rheumatology, Chiron Medical Group, 26/F, 9 Queen’s Road, Central, Hong Kong China

**Keywords:** Bechet’s disease, Colitis, anti-IL 17, anti-TNF, Gastrointestinal

## Abstract

**Background:**

Behcet’s disease (BD) is a systemic disease characterized by recurrent oral and genital ulcers. The underlying disease pathway likely involves interleukin (IL)-17 A, a proinflammatory cytokine that is implicated in Behcet’s uveitis. Secukinumab is an anti-IL-17 A drug that may have an emerging role in the treatment of refractory BD. This is the first known case report of gastrointestinal BD flare up after anti-IL-17 A therapy.

**Case presentation:**

We presented a case of BD with cutaneous and articular features being treated with secukinumab. After the third dose of loading secukinumab, the patient developed acute lower abdominal pain required hospital admission. Urgent computer tomography (CT) abdomen showed fatty stranding of caecum. Colonoscopy with caecal showed increased number of inflammatory cells in lamina propria. Secukinumab was stopped and patient was started on medium dose steroid. His abdominal symptoms resolved after treatment.

**Conclusions:**

This case report illustrates a case of gastrointestinal (GI) BD presenting as acute inflammatory colitis after the use of secukinumab. Therefore, anti-IL-17 A agents should be used cautiously in patients with GI BD, and preferably guided by a phenotype-tailored approach.

## Background

Behcet’s disease (BD) is a systemic disease characterized by recurrent oral and genital ulcers. The underlying disease pathway likely involves IL-17 A, a proinflammatory cytokine that is implicated in Behcet’s uveitis. Secukinumab is an anti-IL-17 A drug that may have an emerging role in the treatment of refractory BD. Conventionally used in autoimmune diseases such as ankylosing spondylitis (AS) and psoriatic arthritis (PsA), secukinumab has efficacy for mucosal and articular manifestations in treatment-resistant BD phenotypes. However, it may worsen or even induce de novo inflammatory bowel disease (IBD). We reported a case of gastrointestinal BD flare up after anti-IL-17 A therapy.

## Case report

**Fig. 1:**
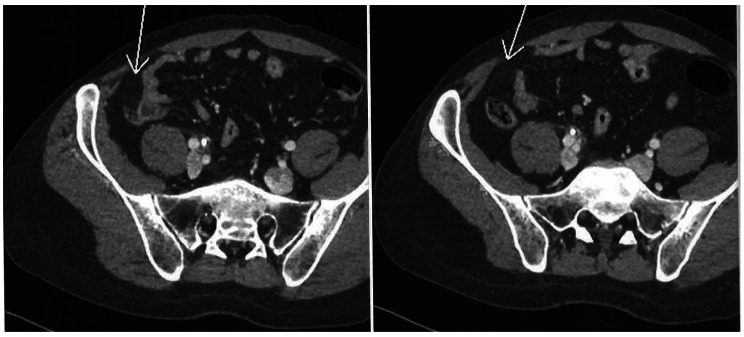
Mild fatty stranding at right lower quadrant of the abdomen, anterior to the cecum and adjacent to the distal ileum (shown by arrows). Features could be related to mild colitis/ ileitis

**Fig. 2:**
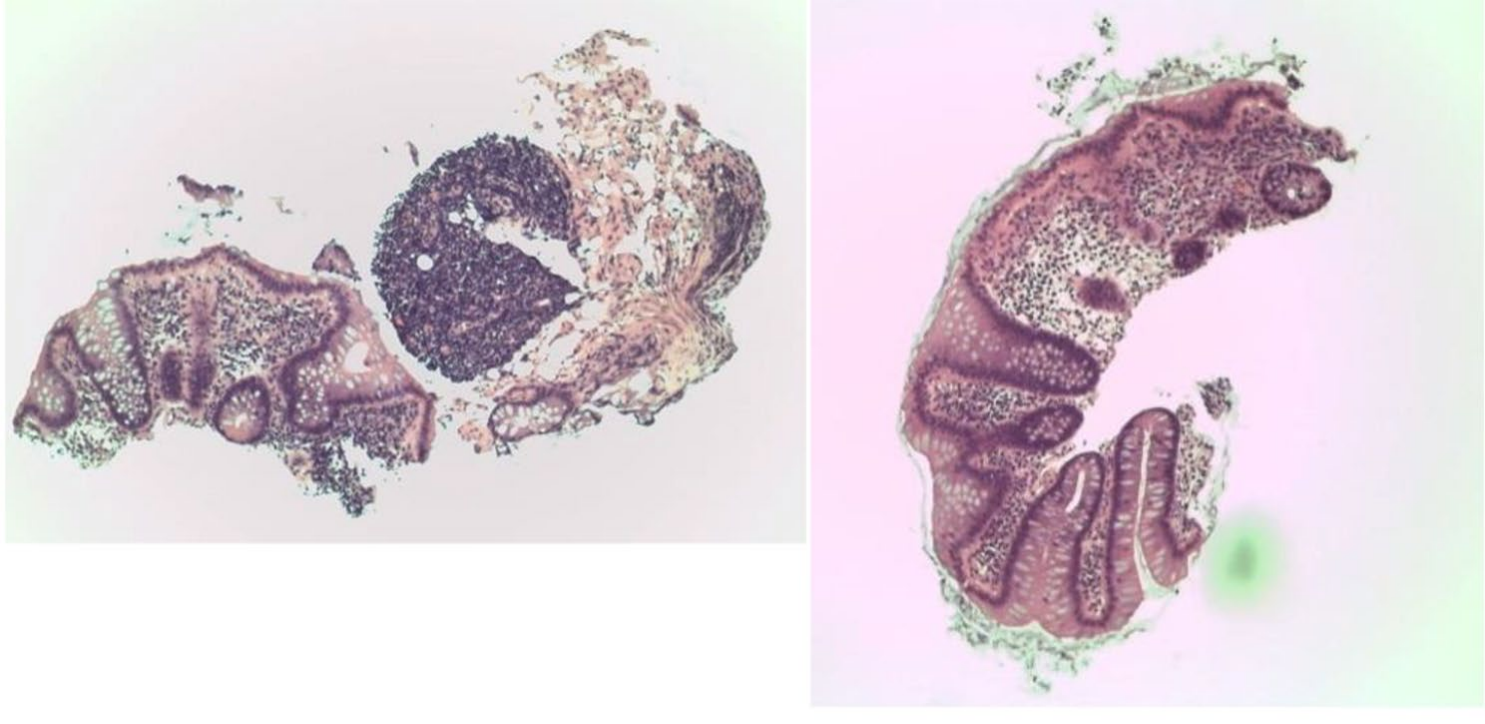
Two pieces of caecal biopsy specimens showing increased number of inflammatory cells in lamina propria

A 34-year-old gentleman with history of renal stones and migraine presented to the rheumatology clinic with recurrent oral and genital ulcers, extensive folliculitis, epididymitis, and polyarthritis for six months. There was no history of uveitis. A recent colonoscopy done for a symptom of rectal bleeding was normal. Physical examination showed multiple oral and genital ulcers, folliculitis on the neck and back, and tender and swollen wrists and knees. Blood tests for anti-nuclear antibody (ANA), and anti-extractable nuclear antigens (anti-ENA) were negative. Complements 3 and 4 (C3 and C4) were normal. Human leucocyte antigen (HLA) B51 was positive and HLA B27 was negative. HLA A26 was not performed. Ophthalmologic assessment showed no features of uveitis. He was diagnosed with Behcet’s disease (BD) according to the International Study Group (ISG) criteria for Behcet’s [1] and treated with prednisolone 30 mg and azathioprine 50 mg daily. Colchicine 0.5 mg daily had also been started but was stopped due to intolerable diarrhea. After a month on azathioprine, he developed deranged liver enzymes with alanine transaminase (ALT) and aspartate transaminase (AST) greater than three times the upper normal limit of normal. Azathioprine was switched to adalimumab (anti-tumor necrosis factor [anti-TNF], Amgevita) 40 mg once every 2 weeks. Despite two months of combination therapy of steroid and adalimumab, he had persistently active arthritis that impaired daily functioning. Adalimumab was switched to secukinumab (an anti-interleukin [IL] 17a) 150 mg once per week as loading dose after a course of intravenous immunoglobulin (IVIg) therapy (24gram for 5 consecutive days, a total of 2gram per kilogram). The oral-genital ulcers, folliculitis, and polyarthritis resolved. However, after the third dose of secukinumab, he developed acute right lower abdominal pain that required hospital admission. There was no fever, oral ulcers, diarrhea, rectal bleeding, joint pain or new folliculitis. Physical examination showed tenderness and guarding at the right lower quadrant of the abdomen. Investigations showed increased white cell count of 12.3 × 10^9/L (normal 3.7-9.2 × 10^9/L) and neutrophils 10.67 × 10^9/L (normal 1.70-5.80 × 10^9/L). Liver and renal function tests were normal with serum albumin decreased to 31.7 g/L (normal 35.0-52.0 g/L). Erythrocyte sedimentation rate (ESR) was increased from 2 mm/hr to 38 mm/hr. Urine culture, chest x-rays and procalcitonin were normal. Abdominal x-rays showed no fluid level or dilated loops of bowel. Urgent computed tomography (CT) of the abdomen showed mild fatty stranding at right lower quadrant, anterior to the caecum and adjacent to the distal ileum – features suspicious of active colitis (Fig. 1). Stool culture was negative. Colonoscopy with caecal biopsy showed mild increased number of inflammatory cells in lamina propria (Fig. 2).

He was diagnosed with active gastrointestinal (GI) BD. Secukinumab was stopped. Three days of pulsed methylprednisolone (250 mg IV once every 24 h) were followed by the resumption of prednisolone 30 mg daily. Abdominal symptoms were resolved one week later. He was started on golimumab (an anti-TNF) 50 mg once per month with slow taper of prednisolone. His condition remained stable with no recurrence of abdominal pain, and ESR normalized to a value of 21. He continued to follow up in the rheumatology clinic to monitor treatment progress.

## Discussion and conclusions

IL-17 A is a proinflammatory cytokine that is primarily produced by T helper 17 cells and is implicated in many autoimmune diseases such as spondyloarthitis and psoriasis [2, 3]. Elevated serum levels of IL-17 have been found in patients with Behcet’s uveitis, and recurrent aphthous ulcers [4, 5]. BD and spondyloarthritis has been proposed to share a common immunopathogenic background, with major histocompatibility complex class I (MHC-I) molecules and the IL-17 axis as crucial components [4, 6]. Secukinumab is a fully human monoclonal antibody against IL-17 A that is used as an effective treatment in psoriatic arthritis (PsA) and ankylosing spondylitis (AS) [7], and shows promising results in the treatment of BD. A multi-centre study suggested that secukinumab at a dose of 150–300 mg per month is effective and with no serious adverse effects in the long-term management of mucosal and articular phenotypes that are resistant to traditional treatments [8].

However, anti-IL-17 A agents has been associated with flares in inflammatory bowel disease (IBD). IL-17 mediates the tight-junction formation of intestinal epithelial cells via a modulation of claudin expression [9]. Therefore, blockage of IL-17 could worsen colitis. In a randomized control study of patients with Crohn’s disease (CD), the use of secukinumab caused more exacerbations compared to placebo [10]. Another multicenter phase II trial demonstrated high rate of serious adverse events and fungal infections [7]. There have been reports of de novo CD in patients with psoriasis and AS while on secukinumab [11]. This poses a difficulty as GI BD and IBD, in particular CD, share many similarities in patient demographics, inflammatory markers, and multiorgan involvement [12]. As a result, they are often classified under the same disease entity. Approximately 10–15% of patients with BD have GI BD, defined by symptoms of colic and diarrhea and signs of typical ulcerations on endoscopy [13]. GI BD typically has poor prognosis due to chronicity of symptoms and poor respond to conventional therapy.

This case report illustrated the potential risk of BD-associated GI flare triggered by the use of secukinumab, possibly from a similar mechanism to IBD. Although secukinumab is promising and has been shown to be safe and effective for the treatment of severe mucosal and articular disease, its use in GI BD requires further review. Secukinumab should be used with caution in GI BD, and guided by a phenotype-tailored approach to therapy. Further research in this area could help us to identify the safeness of IL-17 antagonist in BD.

## Data Availability

Data is available from Dr Ho Yin Chung upon reasonable request.

## Abbreviations

BD: Behcet’s disease
IL: Interleukin
CT: Computer tomography
AS: Ankylosing spondylitis
PsA: Psoriatic arthritis
IBD: Inflammatory bowel disease
ANA: Anti-nuclear antibody
ENA: Extractable nuclear antigens
HLA: Human leucocyte antigen
C3 and C4: Complements 3 and 4
ALT: Alanine transaminase
AST: Aspartate transaminase
TNF: Tumor necrosis factor
ESR: Erythrocyte sedimentation rate
GI: Gastrointestinal
CD: Crohn’s disease

## References

[1] Mizushima Y (1988). Recent research into Behçet’s disease in Japan. Int J Tissue React.

[2] Jin W, Dong C (2013). IL-17 cytokines in immunity and inflammation. Emerg Microbes Infect.

[3] Deodhar A (2019). Long-term safety of secukinumab in patients with moderate-to-severe plaque psoriasis, psoriatic arthritis, and ankylosing spondylitis: integrated pooled clinical trial and post-marketing surveillance data. Arthritis Res Ther.

[4] Nanke Y, Yago T, Kotake S. *The Role of Th17 Cells in the Pathogenesis of Behcet’s Disease*. J Clin Med, 2017. 6(7). 10.3390/jcm6070074 PMC5532582 28753995

[5] Dincses E (2019). Secukinumab induced Behcet’s syndrome: a report of two cases. Oxf Med Case Reports.

[6] Na SY, Park M-J, Park S (2013). Up-regulation of Th17 and related cytokines in Behcet’s disease corresponding to disease activity. Clin Exp Rheumatol.

[7] Koenders MI, van den Berg WB (2016). Secukinumab for rheumatology: development and its potential place in therapy. Drug Des Devel Ther.

[8] Fagni F (2020). Long-term effectiveness and safety of secukinumab for treatment of refractory mucosal and articular Behcet’s phenotype: a multicentre study. Ann Rheum Dis.

[9] Ogawa A, Andoh A, Araki Y, Bamba T, Fujiyama Y. Neutralization of interleukin-17 aggravates dextran sulfate sodium-induced colitis in mice. Clin Immunol. 2004 Jan;110(1):55–62. 10.1016/j.clim.2003.09.013 14962796

[10] Hueber W (2012). Secukinumab, a human anti-IL-17A monoclonal antibody, for moderate to severe Crohn’s disease: unexpected results of a randomised, double-blind placebo-controlled trial. Gut.

[11] Fobelo Lozano MJ, Serrano Gimenez R, Castro Fernandez M (2018). Emergence of inflammatory bowel Disease during Treatment with Secukinumab. J Crohns Colitis.

[12] Valenti S (2017). Intestinal Behcet and Crohn’s disease: two sides of the same coin. Pediatr Rheumatol Online J.

[13] Yazisiz V (2014). Similarities and differences between Behcet’s disease and Crohn’s disease. World J Gastrointest Pathophysiol.

